# Correction to *Lancet Child Adolesc Health* 2018; 2: 569–81

**DOI:** 10.1016/S2352-4642(18)30366-3

**Published:** 2019-01

*Gaccioli F, Sovio U, Cook E, Hund M, Charnock-Jones DS, Smith GCS. Screening for fetal growth restriction using ultrasound and the sFLT1/PlGF ratio in nulliparous women: a prospective cohort study.* Lancet Child Adolesc Health *2018;* 2: *569–81*—The Copyright line of this Article has been updated to reflect that the paper is now Open Access, under the CC BY 4.0 license. This correction has been made to the online version as of Nov 12, 2018.

